# Neuroprotective efficacy of P7C3 compounds in primate hippocampus

**DOI:** 10.1038/s41398-018-0244-1

**Published:** 2018-09-26

**Authors:** Melissa D. Bauman, Cynthia M. Schumann, Erin L. Carlson, Sandra L. Taylor, Edwin Vázquez-Rosa, Coral J. Cintrón-Pérez, Min-Kyoo Shin, Noelle S. Williams, Andrew A. Pieper

**Affiliations:** 1 Department of Psychiatry and Behavioral Sciences, University of California, Davis, USA; 2 UC Davis MIND Institute, University of California, Davis, USA; 3 California National Primate Research Center, Davis, USA; 4 Department of Public Health Sciences, University of California, Davis, USA; 5 University Hospital Case Medical Center; Department of Psychiatry Case Western Reserve University; Geriatric Research Education and Clinical Centers, Louis Stokes Cleveland VAMC, Harrington Discovery Institute, Cleveland, OH 44106 USA; 6 UT Southwestern Medical Center, Department of Biochemistry, Dallas, TX USA

## Abstract

There is a critical need for translating basic science discoveries into new therapeutics for patients suffering from difficult to treat neuropsychiatric and neurodegenerative conditions. Previously, a target-agnostic in vivo screen in mice identified P7C3 aminopropyl carbazole as capable of enhancing the net magnitude of postnatal neurogenesis by protecting young neurons from death. Subsequently, neuroprotective efficacy of P7C3 compounds in a broad spectrum of preclinical rodent models has also been observed. An important next step in translating this work to patients is to determine whether P7C3 compounds exhibit similar efficacy in primates. Adult male rhesus monkeys received daily oral P7C3-A20 or vehicle for 38 weeks. During weeks 2–11, monkeys received weekly injection of 5′-bromo-2-deoxyuridine (BrdU) to label newborn cells, the majority of which would normally die over the following 27 weeks. BrdU+ cells were quantified using unbiased stereology. Separately in mice, the proneurogenic efficacy of P7C3-A20 was compared to that of NSI-189, a proneurogenic drug currently in clinical trials for patients with major depression. Orally-administered P7C3-A20 provided sustained plasma exposure, was well-tolerated, and elevated the survival of hippocampal BrdU+ cells in nonhuman primates without adverse central or peripheral tissue effects. In mice, NSI-189 was shown to be pro-proliferative, and P7C3-A20 elevated the net magnitude of hippocampal neurogenesis to a greater degree than NSI-189 through its distinct mechanism of promoting neuronal survival. This pilot study provides evidence that P7C3-A20 safely protects neurons in nonhuman primates, suggesting that the neuroprotective efficacy of P7C3 compounds is likely to translate to humans as well.

## Introduction

Novel therapeutics are severely lacking for patients suffering from neuropsychiatric and neurodegenerative diseases^1–4^. One promising avenue for central nervous system (CNS) drug development is to address alterations in the magnitude of postnatal hippocampal neurogenesis and hippocampal volume reported for many CNS disorders, including Alzheimer’s disease, schizophrenia, major depression, addiction and anxiety^5,6^. Although questions have recently been raised regarding the extent of adult human hippocampal neurogenesis^7,8^, converging evidence from human and animal studies suggests that the ability to augment the net magnitude of postnatal neurogenesis may present a potential therapeutic intervention for multiple RDoC domains inclusive of symptoms associated with depressive and bipolar disorders, as well as for general conditions of impaired cognition^9–15^. A rigorous preclinical research pipeline is needed to translate promising pharmacological interventions identified in animal model systems into new therapeutic interventions^16^.

Here we present a novel, cross-species approach to advancing preclinical evaluation of the aminopropyl carbazole compound P7C3-A20 from rodents to nonhuman primates. In rodents, the P7C3-series of compounds enhances neuronal survival under conditions that would normally lead to cell death^3^. The prototypical P7C3 molecule was first discovered through an unbiased in vivo screen for drug-like molecules capable of safely enhancing the net magnitude of postnatal hippocampal neurogenesis in mice^17^. The third compound (C3) of the seventh pool (P7), thereby named P7C3, was discovered to have this ability by virtue of protecting young hippocampal neurons from dying, without affecting their rate of proliferation in the postnatal hippocampus. Aged rats that underwent prolonged administration of P7C3 also performed better on cognitive tasks and exhibited decreased cell death in the hippocampus^17^. Subsequently, the neuroprotective effects of P7C3 and its derivative compounds have been further demonstrated in broad preclinical rodent models of CNS disease and injury, including environmental stress-related hippocampal cell death^18–20^, amyotrophic lateral sclerosis^21^, Parkinson’s disease^22–25^, traumatic brain injury^26–28^, peripheral nerve crush^29^, chemotherapy-induced peripheral neuropathy^30^, optic nerve injury^31,32^, Alzheimer’s disease^33^, stroke^34–36^, and recently expanded to acetaminophen-induced liver toxicity^37^. Mechanistically, P7C3 increases nicotinamide adenine dinucleotide (NAD) flux in mammalian cells under conditions of otherwise overwhelming energy crisis that would normally lead to cell death^38^.

While preclinical rodent models have laid the foundation for our understanding of the neuroprotective efficacy of P7C3 compounds, there are limitations in relying solely on rodent models to develop novel therapeutics for complex CNS diseases in humans^39^. Moreover, completion of all stages of adult hippocampal neurogenesis (i.e., proliferation, differentiation, survival, and integration) takes several weeks in rodents^40^, but requires months longer in both human^41^ and nonhuman primates^42^. Therefore, preclinical evaluation of drugs targeting hippocampal neurogenesis may benefit from experiments in animals more closely related to humans, such as the rhesus macaque monkey (*Macaca mulatta*). Rhesus monkeys are genetically and physiologically similar to humans, and are the most widely used nonhuman primate in biomedical research^43^. Moreover, the complex neuroanatomy and behavioral repertoire of the rhesus monkey provides a powerful preclinical platform to evaluate promising CNS therapeutic agents^44,45^. As an initial step in establishing a robust nonhuman primate model, we first evaluated the neuroprotective efficacy of prolonged (38 week) oral administration of P7C3-A20, one of the most highly-potent compounds in the P7C3 series^20,21,23,26,35^, on hippocampal neurogenesis in adult, male rhesus monkeys. Monkeys from P7C3-A20 treatment and vehicle control groups received weekly injections of the thymidine analog cell-division marker, 5′-bromo-2-deoxyuridine (BrdU) during weeks 2–11 of compound exposure, followed by an extended period of 27 weeks of either P7C3-A20 or vehicle administration. In parallel, we also executed a study in mice to directly compare the proneurogenic efficacy of P7C3-A20 to that of NSI-189, a proneurogenic drug currently in clinical trials for patients with major depression. Here we present our initial findings using this integrated in vivo mouse-to-monkey evaluation of P7C3 compounds.

## Materials and methods

Methods for the nonhuman primate and rodent studies are summarized briefly below and described in detail in the supplemental material.

### Nonhuman primate studies

#### Animal selection

Experimental procedures were developed in consultation with the veterinary staff at the California National Primate Research Center and performed in accordance with the University of California, Davis Institutional Animal Care and Use Committee. Eight adult male rhesus monkeys (*Macaca mulatta*) were randomly assigned to one of two treatment groups: (i) Treatment with P7C3-A20 or (ii) Vehicle controls (Table 1). While this is modest sample size for a pilot study, a target sample size of *N* = 4 per group is not uncommon in nonhuman primate research^46,47^. Moreover, previous P7C3A20 data in rodent models demonstrated sufficiently low variability to achieve statistical significance with groups of this size. Unfortunately, one of the vehicle control animals was flagged by veterinary staff for health related concerns and was removed from plans for BrdU quantification resulting in a final sample of *N* = 4 P7C3A20 treated and *N* = 3 vehicle control.

**Table 1:** Experimental groups

Treatment	Age (years)	Weight (kg)
P7C3-A20 (*N* = 4)	5.71	11.56
Vehicle (*N* = 4)^a^	5.98	11.91

^a^One vehicle treated control was removed from BrdU quantification for unrelated medical issues, but data was included for pharmacokinetic analyses and tissue toxicity studies

#### P7C3-A20 formulation and treatment

**Fig. 1:**
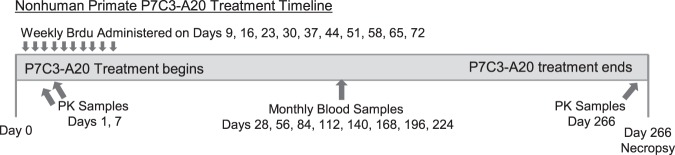
Experimental timeline

P7C3-A20 was synthesized under GLP conditions by Southwest Research Institute as further described in supplemental material. Daily oral administration of compound (10 mg/kg, 0.5 ml/kg) or vehicle control (0.5 ml/kg) was implemented at 9 am (+/−30 min) for 266 consecutive days (38 weeks) by experienced animal care technicians (Fig. 1). General health and appetite were monitored daily by trained technicians and/or veterinary staff.

#### BrdU injections

Each monkey received weekly injections of the thymidine analog cell-division marker, 5′-bromo-2-deoxyuridine (BrdU; Boehringer Mannheim, 150 mg/kg i.v.) on treatment Day 9, 16, 23, 30, 37, 44, 51, 58, 65, and 72 between 1 and 3 pm in the afternoon. The dose of BrdU was selected in order to maximize the amount of labeling, while minimizing the possible toxic side-effects of the marker^48^. The final BrdU injection on Day 72 occurred 194 days (27 weeks) prior to euthanasia. Previous groups have utilized an interval of 2.5 months between BrdU injection and euthanasia to allow sufficient time for nonhuman primate cells to mature^49^. However, more recent evidence indicates that granule cell maturation in the nonhuman primate is protracted over a minimum 6 month time period^42^. For this reason, we extended the time between the final BrdU injection (day 72) and euthanasia (day 266) to 6.5 months^50,51^.

#### Pharmacokinetic analysis

Collection of baseline (pre-treatment) and monthly blood samples are described in supplemental materials and Fig. 1. Blood samples were processed for plasma (PK samples) or both plasma and serum (Monthly samples). CBC/CHEM panels were run on Day 0 (pre-treatment), Day 7 and at each monthly blood sample. Detection of P7C3-A20 in plasma of monkeys was conducted by Abbvie Pharmaceuticals (North Chicago, IL, USA) following a protocol previously developed for evaluation of P7C3-A20 in rodent samples)^27^. Compound extraction of plasma samples is further detailed in supplemental materials.

#### Histology and stereological analyses

Brain collection and processing from monkey followed previously established protocols^52,53^ and is described in further detail in supplemental material. Following perfusion, brains were extracted, placed in refrigerated paraformaldehyde, cryoprotected, and frozen in isopentane. Additional tissues, as described in supplemental materials, were collected and processed for toxicology assessment. The brain was cut into coronal sections with a freezing microtome into six 30μm series and one series at 60 μm (Microm HM 450). Every fourth section of one of the 30μm series (~15 sections evenly-spaced every 960 μm throughout the entire rostrocaudal extent of the hippocampus) was processed with BrdU immunohistochemistry. Additional sections adjacent to the BrdU immunostained sections were stained with a thionin-based Nissl protocol for anatomical reference as previously described^52^. An investigator blind to the treatment status of each specimen performed all analyses, as described below.

The granule cell layer of the dentate gyrus of the hippocampus in both right and left hemispheres was delineated according to previously published cytoarchitectonic descriptions^54–56^. Contours were drawn on each 30 μm BrdU immunostained section (evenly spaced every 960 μm throughout the entire rostrocaudal extent of the hippocampus) and verified with the nissl-stained section contours to provide a consistent boundary for counting the number of BrdU+ cells within the granule cell layer. All measurements were made using Stereoinvestigator software (MBF Bioscience, Williston, VT) on a Zeiss Axio Imager.Z2 Vario microscope with automated stage controller (Ludl Mac6000) and Heidenhan length gage for *z*-axis (section thickness) measurement. Volume measurements were measured according to the Cavalieri principle^57^. The stereological method optical fractionator (counting frame: 1 µm, guard zone: 2 µm) was used to estimate the number of BrdU+ cells. BrduU+ cells were not counted if they came into focus outside of the counting frame (i.e., in the guard zone). The stereological method applied in this study adhered to the sampling scheme specifically described in West^58^, as follows: Given the low number of BrdU+ cells present within the counting frame, the entire area of the section for the disector was used to count all BrdU+ cells that fell within the counting frame (area sampling fraction [asf] = 1) and multiplied by the inverse of the section sampling fraction to obtain estimates of the total number of BrdU+ cells in the granule cell layer^58^.

#### Statistics

We compared mean cell counts between monkeys treated with P7C3-A20 using a two-sample *t*-statistic assuming unequal variances. One animal had a large cell count resulting in a skewed distribution. We therefore used a complete permutation null distribution to determine the significance level, because it does not require assuming the data were drawn from a normally-distributed population. In small samples such as represented in this study, the sample mean can be strongly impacted by very high or low values. Because of the relatively large cell counts for one animal in the intervention group, we also conducted a Wilcoxon rank sum test to compare distributions between the two groups. An exact *p*-value was calculated for this test. All tests were two-sided.

### Rodent studies

All rodent animal procedures were performed in accordance with the University of Iowa animal care committee’s regulations. Animals were housed in temperature-controlled conditions, provided food and water ad libitum, and maintained on a 12 h light/dark cycle (7:00 am–7:00 pm). Male C57BL/6 J mice were purchased from The Jackson Laboratory. Experiments designed to compare P7C3-A20 to NSI-189 for ability to increase the net magnitude of hippocampal neurogenesis using a standard 5 day in vivo assay were followed by targeted assays of proliferation and survival and newborn hippocampal neurons in the mouse dentate gyrus (described in supplemental material). General health and appearance of all animals was monitored daily by trained technician and veterinary staff, and no abnormalities were noted during this brief treatment period. After 5 days of daily BrdU (Sigma-Aldrich) administration at 9:00 am, mouse brains were collected and prepared as described in supplemental materials. The number of BrdU+ cells in the entire dentate gyrus subgranular zone (SGZ) was quantified per our established procedures^17,19,20,23,24,59^. Briefly, BrdU+ cells are manually counted under 20× magnification within the SGZ and dentate gyrus in every fifth section throughout the entire hippocampus. Then a picture of the dentate gyrus field is obtained at 4X magnification, and the volume of the dentate gyrus granular cell layer is determined by using NIH Image J software tailored to the microscope to trace the area, and then multiplying by the thickness of the section. All data were normally distributed; therefore, in instances of multiple mean comparisons, analysis of variance was used, followed by post hoc comparison using Tukey’s method. In instances of direct comparison, two-tailed Student’s *t*-test was performed. Alpha levels were set to .05, and analyses were conducted using GraphPad Prism (GraphPad Software, Inc., La Jolla, CA). The presence our outliers was tested using ROUT method in Prism and defined as having *Q* = 0.1%. In this study, no outliers were detected. Significance is denoted as *p = .05, **p = .01, ***p = .001, ****p = .0001, and not significant. A blinded examiner conducted all analyses, and code was not broken until analyses were completed.

## Results

### 38 Weeks of daily oral dosing (10 mg/kg) of P7C3-A20 provides sustained plasma exposure in nonhuman primates

**Fig. 2:**
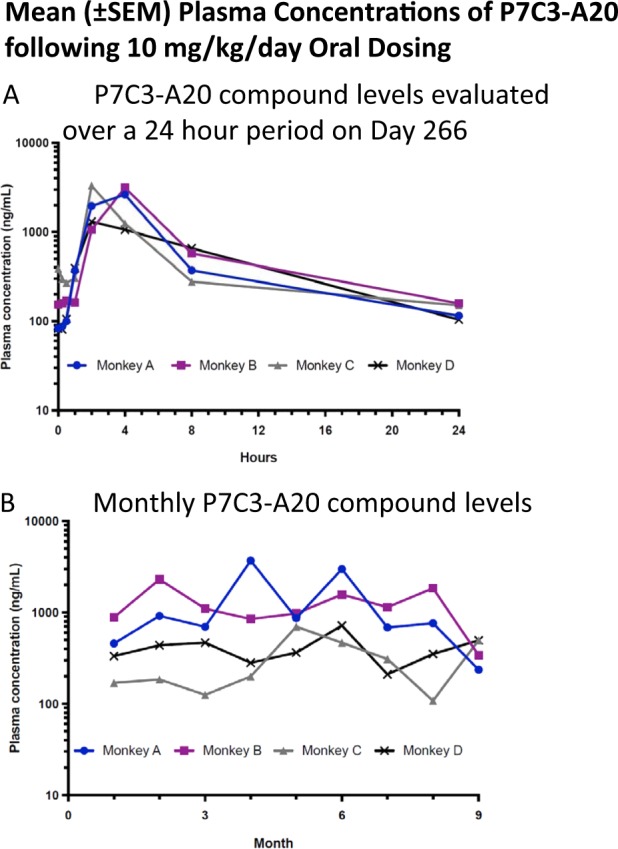
a P7C3-A20 compound levels were evaluated over a 24 h period on Days 1, 7, and 266, and the overall exposure measured (area under the curve: AUC) differed by no more than two-fold. Compound levels varied from an average Cmax of 963 to 2320 ng/ml at around 4 h to a trough of 73–132 ng/ml at 24 h immediately prior to the next dose. Individual animal data from day 266 are presented in **a**. **b** P7C3-A20 levels were also measured once a month, 6 h after dosing, a time point close to the estimated maximal concentration point

Orally-administered P7C3-A20 provided sustained plasma exposure in rhesus monkeys (Fig. 2). To ensure good compliance for compound administration, a formulation of corn oil and flavored syrup was utilized for oral dosing in the monkeys. Prior to initiation of the primate study, this formulation was evaluated in male Fisher 344 rats to ensure comparable exposures with the rodent DMSO/cremophor formulation previously utilized^21,23^. Exposures were found to be comparable for a 10 mg/kg dose administered by oral gavage (DMSO/Cremophor: AUC 12958 h*ng/ml, *C*_max_: 928 ng/ml; Oil/Syrup: AUC 15174 h*ng/ml, *C*_max_ 1155 ng/ml). Drug levels were evaluated over a 24 h period on Day 1, 7, and 266. Overall exposure, defined as area under the curve (AUC), differed by no more than two-fold. Compound levels varied from an average *C*_max_ of 963–2320 ng/ml at around 4 h, to a trough of 73–132 ng/ml at 24 h just prior to the next dose, as presented at day 266 (Fig. 2a). P7C3-A20 levels were also measured once a month, 6 h after dosing, a timepoint close to the estimated maximal concentration point. Here again, levels were fairly consistent and showed no trend towards increase or decrease, supporting the favorable chemical property of lack of induction or inhibition of any clearance mechanisms, such as cytochrome P450-mediated metabolism (Fig. 2b). Finally, although this represents a small cohort of animals, there was little variability between animals, indicating that consistent and fairly equivalent exposures were achieved in each non-human primate.

### Chronic P7C3-A20 ingestion is not associated with toxicity in nonhuman primates

After 38 weeks of daily oral exposure at 10 mg/kg dose of P7C3-A20, tissues were comprehensively collected at necropsy and evaluated by a pathologist blind to experimental condition. No microscopic evidence of toxicology was detected in any of the tissues examined, including eyes, lung, heart, aorta, tongue, spleen, liver, kidney, adrenal, thyroid, parathyroid, pancreas, stomach, testes, small intestine, large intestine, skeletal muscle, vesicular gland, spinal cord, peripheral nerve, prostate, salivary glands, gall bladder, bone marrow, epididymis, optic nerve, lymph nodes, mammary gland, larynx, skin, trachea, ureter, bone, joint, and pituitary gland. This complete lack of toxicity after daily oral administration of P7C3-A20 for 38 weeks to non-human primates is a favorable indication for the translational potential of the P7C3-series into a safe treatment for patients.

**Fig. 3:**
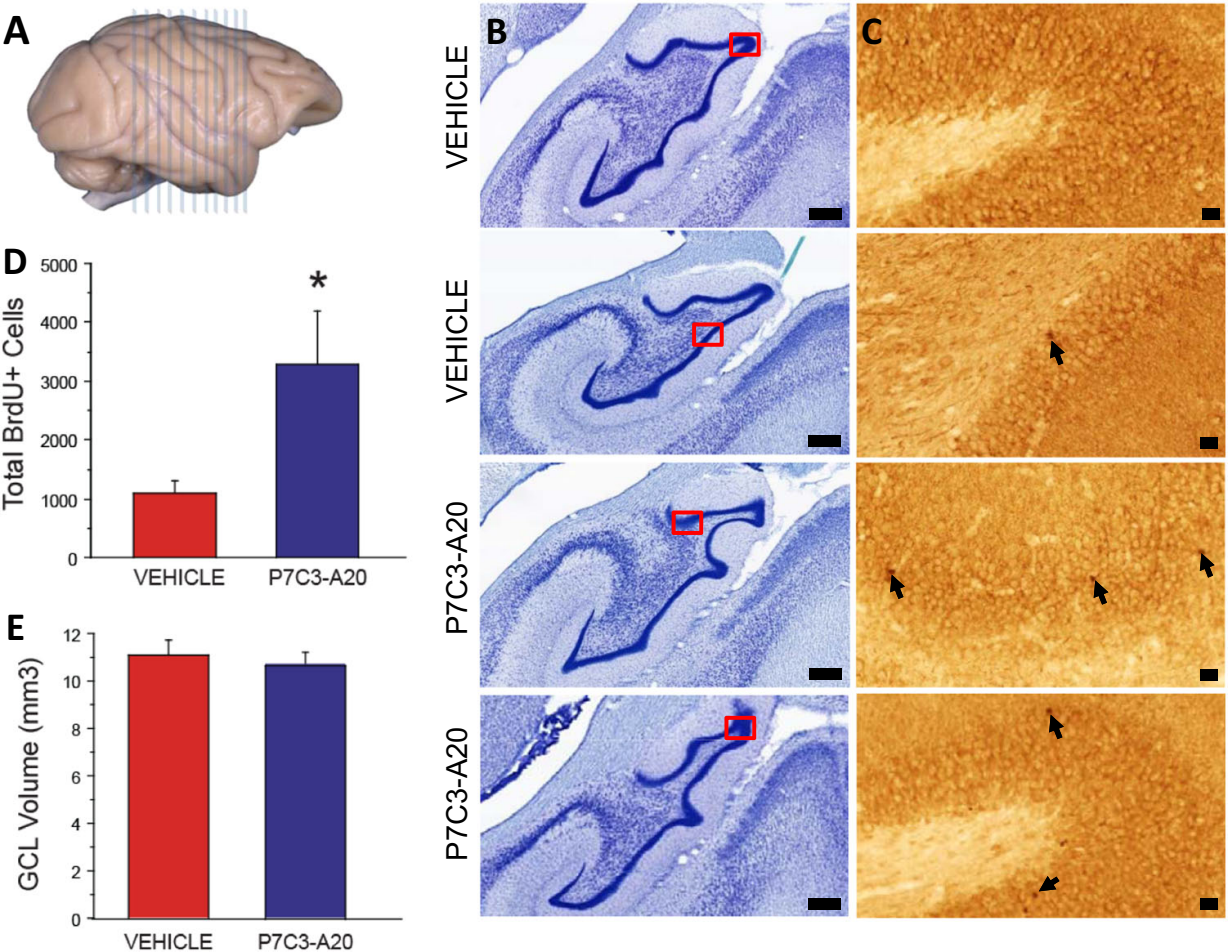
a Approximately 15 sections per animal evenly spaced 960 μm apart covering the rostral to caudal extent of the hippocampus were used to estimate the number of BrdU-labeled cells. **b** Contours were outlined on Nissl-stained sections for anatomical reference. **c** Contours were drawn on BrdU immunohistochemically stained sections to measure volume and count the number of BrdU-labeled cells within the granule cell layer. **d** The total number of BrdU positive cells (left and right hemispheres) differed significantly (*p* = 0.029) between monkeys treated with P7C3A20 (mean = 3275, SD = 1847) and those that received the vehicle control (mean = 1095, SD = 368). However, median values fell just shy of the set significance value at 0.05 level (*p* = 0.056). **e** As predicted, the groups did not differ in bilateral GCL volume (*p* = 0.857) as a function of P7C3-A20 exposure

Orally-administered P7C3-A20 elevates survival of newborn hippocampal neurons in nonhuman primates: Survival of young hippocampal neurons was examined 27 weeks after the final injection of the cell-division marker BrdU (Fig. 3a–c; Table 2). The mean number of BrdU+ cells differed significantly (*t* = *−*2.30, df = 3.31*, p* = 0.029 based on permutation null distribution) between monkeys treated with P7C3-A20 (mean = 3275, SD = 1847) and those that received the vehicle control (mean = 1095, SD = 368) (Fig. 3d). Although the Wilcoxon test comparing the two distributions fell just short of statistical significance based on a 0.05 threshold (*W* = 0, *p* = 0.057), all treated animals had higher numbers of BrdU+ cells than animals in the control group, as shown in (Supplemental Fig. 1). Animals were randomly assigned to treatment group in order to best control for the possibility of inter-animal variation in baseline postnatal neurogenesis, and did not differ in bilateral GCL volume (*p* = 0.857) as a function of P7C3-A20 exposure (Fig. 3e). Although BrdU can also label DNA repair events, this is unlikely to be a factor in our study that showed a difference between P7C3-A20 and vehicle groups, as P7C3 compounds have an extensive record of safety in published preclinical models and we also saw no evidence of toxicity anywhere in the body associated with P7C3-A20 administration.

**Table 2:** Summary of average number BrdU positive cell counts and GCL volumes [Mean (±SEM)]

	BrdU positive cells		GCL volume (mm³)	
	Left hemisphere	Right hemisphere	Left hemisphere	Right hemisphere
Vehicle	597 (±115)	498 (±116)	10.7 (±0.2)	11.4 (±1.3)
P7C3-A20 treated	1495 (±514)	1781 (±409)	10.1 (±0.8)	11.3 (±0.5)

### P7C3-A20 is more efficacious and mechanistically distinct in mice from the proneurogenic drug NSI-189

**Fig. 4:**
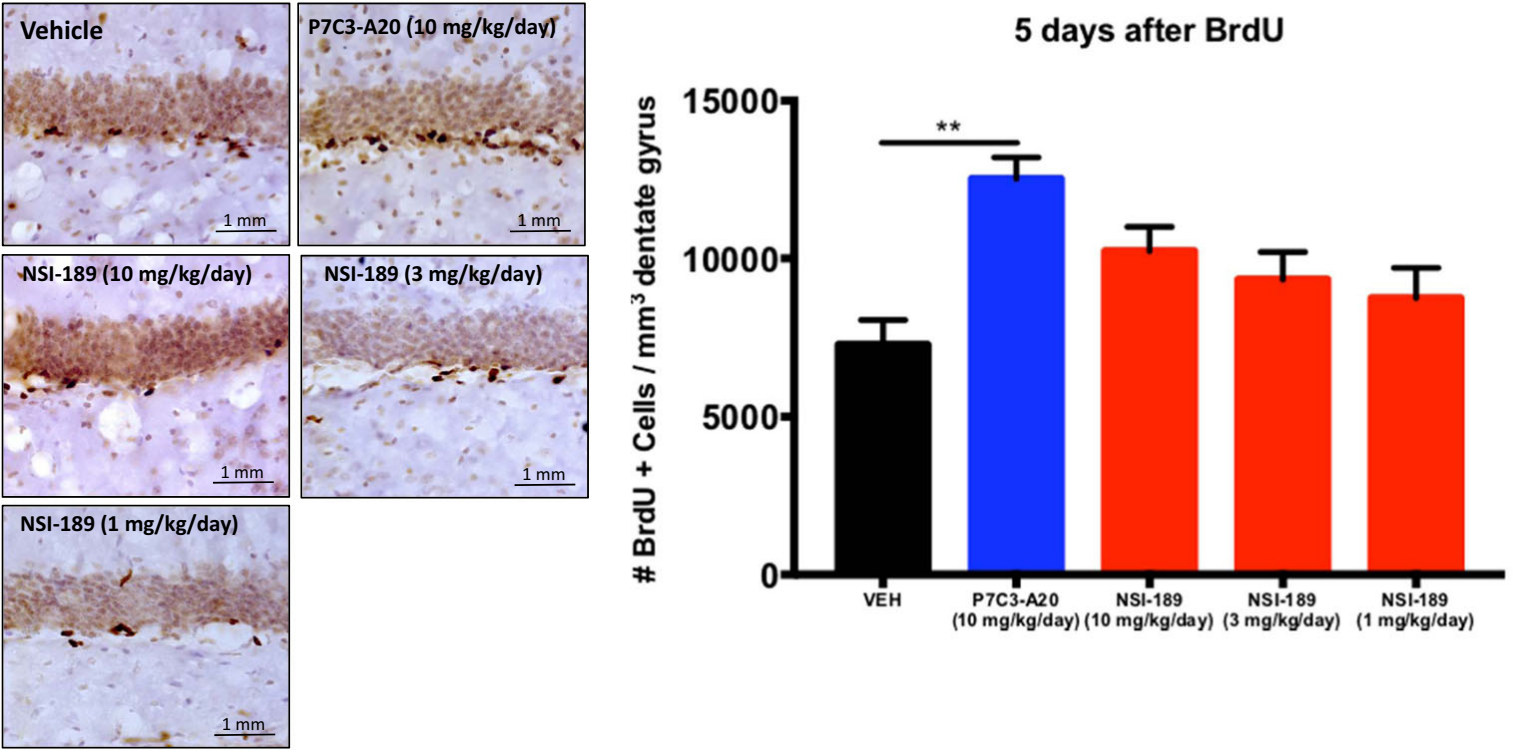
Compounds were administered intraperitoneally at the daily dose indicated for 5 days, during which time mice were also dosed intraperitoneally daily with BrdU (50 mg kg^-1^day^-1^) to label newborn hippocampal neurons. Representative micrographs are shown on the left, with quantified data on the right represented as mean +/- standard error of the mean (SEM). NSI-189-treated mice showed a nonsignificant increase in the number of BrdU+ cells that was statistically not different from vehicle. P7C3-A20-treated mice showed a statistically significant (*p* < .01) increase in the number of BrdU+ cells compared to vehicle-treated animals

We next compared in mice the efficacy of P7C3-A20 to that of NSI-189, an experimental proneurogenic drug that acts by unknown mechanisms to enhance hippocampal neurogenesis. To date, NSI-189 has shown protective efficacy in a rat model of stroke^60^, as well as a promising effect in a phase1B randomized double-blinded placebo-controlled multiple dose-escalation study in adult patients with major depressive disorder^61^. We first compared P7C3-A20 to NSI-189 for the ability to increase the net magnitude of hippocampal neurogenesis in a standard 5 day in vivo assay of BrdU-labeled cells in the dentate gyrus^17^. Here, mice were socially isolated without any environmental enrichment for 2 weeks, per our standard protocol, and then subjected to daily administration of both the test proneurogenic compound and a daily dose of BrdU (50 mg/kg ip). In this experiment, NSI-189 showed a nonsignificant dose-dependent increase in the number of BrdU + cells, whereas P7C3-A20 showed a statistically significant (*p* < .01) increase in the number of BrdU+ cells compared to vehicle-treated animals (Fig. 4).

**Fig. 5:**
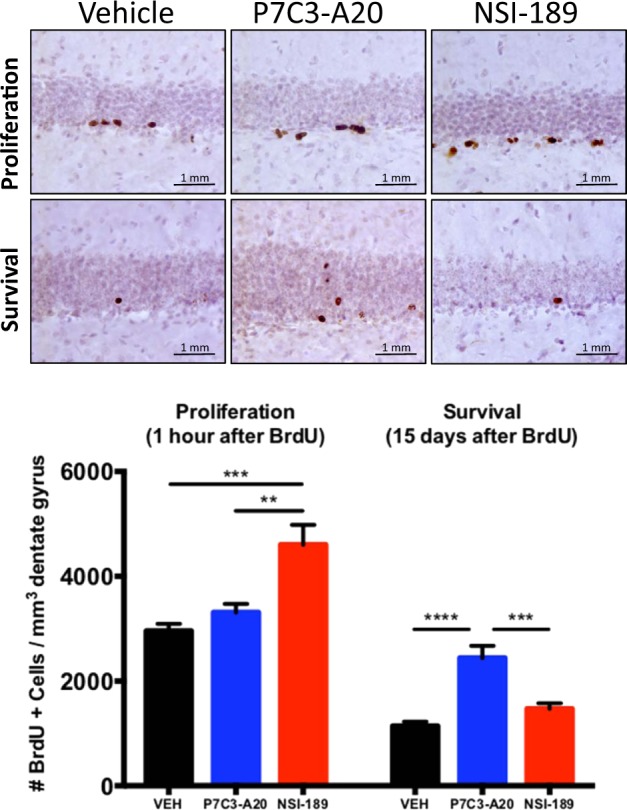
Efficacy on enhancing proliferation of hippocampal neural precursor cells and survival of young hippocampal neurons was compared between NSI-189 and P7C3-A20 at a dose of 10 mg kg^−1^day^−1^ administered intraperitoneally. Representative micrographs of BrdU+ cells in the dentate gyrus are shown above, with quantified data displayed below as mean +/- standard error of the mean (SEM). Proliferation was assayed in animals that had received 3 days of daily P7C3-A20 or NSI-189, followed by a 1 h pulse of of BrdU (150 mg/kg ip) P7C3-A20 elicited no increase in cellular proliferation compared to the level seen in vehicle-treated animals. NSI-189 elevated proliferation of neural precursor cells by approximately 70% relative to vehicle (*p* < .001) and P7C3-A20-treated mice (*p* < 0.01). Neuroprotective efficacy for young hippocampal neurons was assayed in animals that received the same pulse of BrdU at the same time as initiation of 15 days of daily treatment with P7C3-A20 or NSI-189. P7C3-A20-treated animals showed a survival rate approximately three times greater than vehicle-treated (*p* < .0001) and NSI-189-treated (*p* < .001) animals, with migration of some cells out of the subgranular zone and into the dentate gyrus. Animals treated with NSI-189 showed a minor and non-significant trend towards increased survival, indicating that the proneurogenic efficacy of NSI-189 is likely confined to its pro-proliferative effect on cells. P7C3 compounds increase the net magnitude of hippocampal neurogenesis to a greater extent than NSI-189 by virtue of the unique ability of P7C3 compounds to protect neurons from cell death

The standard 5 day assay captures agents that increase the net magnitude of hippocampal neurogenesis, without distinguishing whether they act by enhancing proliferation or promoting survival. For example, this assay was originally used to discover the prototypical P7C3 molecule^17^, which was subsequently shown to elevate hippocampal neurogenesis by selectively blocking neuronal death with affecting proliferation of neural precursor cells. Thus, we compared P7C3-A20 to NSI-189 in standard assays of proliferation (1 h after BrdU administration) and survival (15 days after BrdU administration) (Fig. 5). Proliferation was assayed by measuring the number of BrdU+ cells/mm^3^ dentate gyrus 1 h after a pulse of BrdU (150 mg/kg ip) in animals that had already received 3 days of daily P7C3-A20 (10 mg/kg/day ip) or NSI-189 (10 mg/kg/day ip). Here, P7C3-A20 elicited no increase in cellular proliferation, compatible with our previous observations, while treatment with NSI-189 elevated proliferation of neural precursor cells by approximately 70%. Next, to assay the neuroprotective efficacy for young hippocampal neurons, we measured the number of BrdU+ cells/mm^3^ dentate gyrus 15 days after a pulse of BrdU (150 mg/kg ip) in animals that received daily treatment with either P7C3-A20 (10 mg/kg/day ip) or NSI-189 (10 mg/kg/day ip) starting at the time of delivery of the BrdU pulse. Here, animals treated with P7C3-A20 showed a survival rate approximately three times greater than vehicle-treated animals, with migration of some cells out of the subgranular zone and into the dentate gyrus (Fig. 5). Animals treated with NSI-189, however, showed only a minor and non-significant trend towards increased survival, indicating that the proneurogenic efficacy of NSI-189 is likely confined to its pro-proliferative cellular effect. These experiments show that P7C3 compounds increase the net magnitude of hippocampal neurogenesis to a greater extent than NSI-189 by virtue of the ability of P7C3 compounds to protect neurons from cell death.

## Discussion

P7C3 compounds were first identified through an unbiased screen of hippocampal neurogenesis in rodents^17^, and subsequently shown to have neuroprotective properties for a number of CNS relevant rodent models^18–35^. Here we establish the next step in advancing P7C3 along the translational pipeline into a species more closely related to humans—the rhesus macaque. This study provides two major, novel findings. First, we demonstrate that orally-administered P7C3-A20 provides sustained plasma exposure, is well-tolerated, and protects young hippocampal neurons in the adult male rhesus macaque. A higher number of BrdU+ cells in the dentate gyrus were detected in monkeys treated daily with P7C3-A20 compared to vehicle (Fig. 3). Second, in a direct comparison in mice treated with P7C3-A20 compared to the drug NSI-189 that is currently in clinical trials for patients with major depression, the survival-promoting effect of P7C3-A20 appears to augment the net magnitude of hippocampal neurogenesis more effectively than the pro-proliferative effect of NSI-189 (Figs. 4, 5). The present studies add to a growing body of evidence suggesting that P7C3 compounds offer a promising therapeutic intervention for a number of CNS disorders. Mechanistically, we have shown in rodent models an cellular systems that P7C3 increases neuronal NAD flux under conditions that would normally lead to cell death^38^, and efficacy of P7C3 compounds in the nonhuman primate suggests that this general mechanisms of neuronal protection is likely to be operational in humans as well.

Our initial efforts in the nonhuman primate model focus on the neuroprotective properties of P7C3 compounds related to adult neurogenesis in the primate hippocampus. As first described in rodents^62^, neurons born in the subgranular zone (SGZ) migrate and integrate into the granule cell layer^50,51,63^, a process that takes months longer in nonhuman primates^42^ and humans^41^ than in rodents. Hippocampal neurogenesis peaks at 3 months of age for macaques and remains at an intermediate level between 3 months and at least 1 year of age (roughly equivalent to early childhood)^49^. Adult macaques and humans demonstrate persistent levels of neurogenesis in adulthood^49,64^, though the number of surviving newborn neurons decreases substantially with age^65^. Studies from rodent models indicate that adult-born neurons demonstrate a transient period of excitability and plasticity thought to recapitulate the early stages of fetal and postnatal brain development^66,67^. These properties suggest that adult-born cells may be uniquely positioned to impact hippocampal activity, in spite of their relatively low abundance^40^. Thus, adult-born neurons are thought to enhance function of hippocampal networks^68,69^, though there is no consensus on exact functional contributions. Although new neurons are generated in the adult human dentate gyrus (DG) of the hippocampus^8,63^, the extent to which human hippocampal neurogenesis persists in aging has been debated^7^. While additional research in this fascinating field is clearly warranted, we also stress that P7C3 compounds are proneurogenic by virtue of the fact that their neuroprotective efficacy encompasses both mature and newborn neurons. A compound that protects against neuronal loss is potentially more attractive for clinical development than one that exclusively targets proliferation of newborn neurons in the brain, as a broad and critical role of neuronal loss has been firmly established in a wide variety of neurological and neuropsychiatric conditions.

Dysregulated hippocampal neurogenesis has been linked to a number of neuropsychiatric diseases, including major depressive disorder, bipolar disorder, schizophrenia and anxiety^70–76^. The ability to regulate adult hippocampal neurogenesis may thus provide a route to develop novel treatments for human neuropsychiatric disease^77^, as evidenced by the links between behavioral effects of antidepressants and stimulation of hippocampal neurogenesis in preclinical^78,79^ and clinical studies^80–82^. The current findings support further investigation of the neuroprotective effects of P7C3 compounds in nonhuman primate models of impaired cognition^83–85^ or stress-related behaviors^86,87^. P7C3 compounds have also demonstrated neuroprotective properties in rodent models of nervous system disease and injury that involve neurodegeneration outside of the hippocampus (i.e., amyotrophic lateral sclerosis, Alzheimer’s disease, Parkinson’s disease, traumatic brain injury, stroke, and chemotherapy-induced peripheral neuropathy). Given that nonhuman primate models exist for these disorders, we are now positioned to explore the therapeutic potential of the P7C3 class of molecules in sophisticated nonhuman primate disease models. In future studies with larger number of animals we will also incorporate analysis of brain levels of P7C3 compounds as well, in addition to blood. This integrated in vivo mouse-to-monkey evaluation of neuroprotective compounds may provide important benchmarks for utilizing cross-species animal models and accelerate drug discovery efforts.

In order to assess the survival-promoting effect of P7C3-A20 relative to the drug NSI-189 currently in clinical trials for major depression, we also carried out a direct comparison in mice and found that P7C3-A20 augmented the net magnitude of hippocampal neurogenesis more effectively than NSI-189. That NSI-189 promoted proliferation but failed to show a sustained increase in net magnitude of hippocampal neurogenesis 15 days later suggests that a brief increase in proliferation does not elicit sustained improvement in hippocampal function. However, it is unknown whether prolonged chronic administration of NSI-189 might eventually achieve this objective through chronic elevation of the rate of proliferation of hippocampal neural precursor cells. We have previously shown that P7C3-A20 exerts potent antidepressant efficacy in a rodent model of chronic social defeat stress^20^ in a manner specifically linked to its ability to increase the net magnitude of hippocampal neurogenesis by virtue of its survival-promoting mode of action, and NSI-189 has shown promising results in a phase1B randomized double-blinded placebo controlled multiple dose-escalation study in adults with major depressive disorder^61^. Inasmuch as augmenting hippocampal neurogenesis can be assumed to represent a novel route to treating patients with depression^76,88^, our results of greater potency and efficacy of P7C3 compounds suggest that a drug to emerge from the P7C3 class of neuroprotective molecules might have superior efficacy to NSI-189. In addition, concurrent administration of P7C3 compounds and pro-proliferative compounds like NSI-189 might have a synergistic effect in enhancing hippocampal neurogenesis and treating depression or hippocampal-related cognitive dysfunction in patients suffering from a spectrum of neurodegenerative conditions related to injury, disease, or normal aging.

Collectively, these studies suggest that the neuroprotective property of P7C3 compounds may have broad applicability for a number of human CNS disorders. The primary limitation of the current study is the small sample size of adult nonhuman primates. However, small sample sizes are not uncommon for initial pilot studies in nonhuman primates^46,47^. Given that early translational studies tend to have quite high per-subject costs, initial nonhuman primate studies must balance the potential information gained versus ethical and financial factors^89^. Moreover, the current nonhuman primate study included only male subjects. In rodent preclinical models of disease in which P7C3 compounds have been previously applied, efficacy has correlated with incorporation of surviving and mature neurons into appropriate circuitry of the brain related to behavioral outcome in both males and females. Despite these limitations, this initial nonhuman primate study has generated preliminary data that support further evaluation of P7C3-A20 compounds as a promising treatment for human CNS diseases. In future studies, we will seek to replicate the nonhuman primate findings in a larger sample and also determine whether P7C3 compounds have efficacy in female nonhuman primates. We will also explore hippocampal asymmetry, given that right and left hippocampal volumes differ in the healthy human brain^90,91^ and in CNS diseases^92,93^. In addition, we will pursue more sophisticated physiologic analysis of neuronal survival and incorporation into brain circuitry related to behavioral outcome measures, such as cognitive and mood-like behaviors, and explore the therapeutic potential of the P7C3 class of molecules in sophisticated nonhuman primate disease models. The ultimate goal is to translate basic science into new therapeutic approaches for patients suffering from neuropsychiatric or neurodegenerative conditions for which there are currently sub-optimal or no treatment options.

## Electronic supplementary material

Supplemental methods
