# Supplementary material for: Neuroprotective efficacy of P7C3 compounds in primate hippocampus
Source: Transl Psychiatry. 2018 Sep 26;8:202. doi: 10.1038/s41398-018-0244-1 (PMC6158178; doi:10.1038/s41398-018-0244-1)
Supplement: Supplementary file 1 — Supplemental methods [file 41398_2018_244_MOESM1_ESM.pdf]

<u>Supplemental Materials</u>

Materials and Methods

<u>Nonhuman Primate Studies</u>

P7C3-A20 Formulation. P7C3-A20 was synthesized under GLP conditions by Southwest Research Institute. P7C3-A20 dose was prepared at 20mg/ml in 60% corn oil (Sigma C8267) and 40% syrup (Torani Syrup). Vehicle formulation consisted of 60% corn oil and 40% syrup. The compound/corn oil was vortexed for 5 minutes, and then allowed to sit for 12-24 hours at room temperature. Prior to oral administration, the compound/corn oil was again vortexed to re-suspend in oil and then sonicated for 15 minutes to suspend compound in oil as a slurry. Torani syrup equal to 40% of the total volume was then added and stored in a dark cabinet at room temperature. Prior to drawing up an individual dose, the solution was vigorously mixed for 30 seconds to thoroughly mix syrup and oil layers. Doses were drawn immediately prior to each weekday dosing. Weekend doses were drawn into dosing syringes on Friday. Syringes were inverted to mix well before dosing.

Treatment with P7C3-A20. Daily oral administration of compound (10mg/kg, 0.5ml/kg) or vehicle control (0.5ml/kg) was implemented at 9am (+/-30min) for 266 consecutive days (38 weeks) by experienced animal care technicians. Animals were provided with food rewards at the time of oral administration, and there were no instances of noncompliance.

Pharmacokinetic analysis. Blood samples included (i) Baseline (pre-treatment) samples, (ii) pharmacokinetic (PK) samples and (iii) CBC/CHEM panel samples. (i) Baseline blood samples were collected 7 days and 1 day prior to treatment. (ii) Blood samples (1ml) for PK analyses were collected at 0.25, 0.5, 1.0, 2.0, 4.0, 8.0,and 24 hrs post treatment on Days 1, 7 and 266. Additional blood samples (8ml) were collected monthly at eight time points (Days 28, 56, 84, 112, 140, 168, 196, and 224). Blood samples were processed for plasma (PK samples) or both plasma and serum (Monthly samples), and stored at -80C. (iii) CBC/CHEM panels were run on Day 0 (pre-treatment), Day 7 and at each monthly blood sample. Detection of P7C3-A20 in plasma of monkeys was conducted by Abbvie Pharmaceuticals (North Chicago, IL) following a protocol previously developed for evaluation of P7C3-A20 in rodent samples (1). Compound was extracted from plasma samples or standards prepared by spiking blank rhesus monkey plasma with GLP grade P7C3-A20 by passage over a Phenomenex (Torrence, CA USA) Phree Phospholipid Removal 1 ml tube to remove phospholipids as well as proteins. Briefly, plasma was loaded onto the column and volume of acetonitrile equal to 3-fold the volume of applied plasma and containing formic acid and an internal standard was added. The mixture was pipeted gently twice to mix. A vacuum was applied and the flow-through collected. An additional 3X volume of acetonitrile was applied to wash the column, and the flow through was then added to the original material. Compound levels in

the resulting flow-through were monitored by LC-MS/MS. Pharmacokinetic parameters were determined using the noncompartmental tool in Phoenix WinNonlin (Certara Corp., Princeton, NJ).

Histology and toxicology analyses. Brain processing followed previously established protocols (2, 3) described briefly below. Monkeys were deeply anesthetized by intravenous injection of sodium pentobarbital (50 mg/kg i.v.; Fatal-Plus; Vortech Pharmaceuticals, Dearborn, MI), and then perfused transcardially with 1% paraformaldehyde in 0.1 M phosphate buffer (250 ml/min) for 2 min, followed by 4% paraformaldehyde (250 ml/min) for 10 min, and 4% paraformaldehyde (100 ml/min) for 50 min. Brains were extracted, placed in refrigerated 4% paraformaldehyde for 6 hours, and then cryoprotected in 10% glycerol / 2% DMSO for 24 hours, followed by 20% glycerol / 2% DMSO for 72 hours before being frozen in an isopentane bath chilled with an outer reservoir of dry ice and ethanol for a minimum of 45 minutes. Coronal sections were cut with a freezing microtome into six 30-μm series and one series at 60 μm (Microm HM 450). The 30-μm sections utilized in this study were collected in a tissue collection solution (TCS) consisting of 30% ethylene glycol, 25% glycerin in PB, and kept at –70°C until further processing. Following fixative perfusion, as described above, additional tissues were collected at necropsy and fixed by immersion in 4% formaldehyde solution for at least 24 hours. Tissues were trimmed and embedded in paraffin using standard histology procedures, and routine 6 um hematoxylin and eosin slides were prepared and identified by animal number but not treatment group. Bony tissues were decalcified in a commercially available decalcifier prior to embedding. The following tissues were qualitatively evaluated by a veterinary pathologist blind to experimental condition, including: eyes, lung, heart, aorta, tongue, spleen, liver, kidney, adrenal, thyroid, parathyroid, pancreas, stomach, testes, small intestine, large intestine, skeletal muscle, vesicular gland, spinal cord, peripheral nerve, prostate, salivary glands, gall bladder, bone marrow, epididymides, optic nerve, lymph nodes, mammary gland, larynx, skin, trachea, ureter, bone, joint and pituitary.

BrdU Immunohistochemistry. Approximately fifteen 30μm-thick sections per animal evenly spaced 960μm apart covering the entire rostral to caudal extent of the hippocampus was stained for BrdU using the free-floating method. An additional section from each case was prepared without primary antibody as a negative control. All washes and buffers were prepared in 0.1M phosphate buffered saline pH 7.4 and run at room temperature unless otherwise stated. 30μm thick sections were incubated in 2N HCl for 1 hour and then neutralized in 0.1M borate buffer, pH 8.5 for 15 minutes. Endogenous peroxidase activity was quenched with a 1% H_2O_2 solution for 30 minutes. Non-specific protein cross reactivity was blocked for 1 hour using a 0.3% triton x-100, 5% normal rabbit serum, and 1% bovine serum albumin buffer. An avidin/biotin blocking kit (SP-2001, Vector Laboratories, Burlingame, CA) was used according to manufacturer's specifications. Primary and secondary antibodies were diluted in 0.3% triton x-100 and 2% normal rabbit serum. Sections were incubated in primary antibody, 1:1000 sheep anti-BrdU(20-BS17, Fitzgerald, Acton, MA) overnight at 4C followed by

secondary antibody, 1:227 rabbit anti-sheep IgG biotinylated antibody (BA 6000, Vector Laboratories, Burlingame, CA) for 1 hour. Sections were incubated in a streptavidin biotinylated horseradish peroxidase complex (IH-8106, ImmunoBioScience Corp, Mukilteo, WA) for 45 min, and then treated to an additional round of secondary antibody for 45 minutes and repeated incubation in streptavidin-HRP complex for an additional 30 minutes. Sections were washed with 50mM tris and immunoreactivity was visualized with a 10 minute incubation in 0.5% 3,3′-Diaminobenzidine tetrahydrochloride hydrate (D9015, Sigma-Aldrich, Germany) solution containing 0.04% H_2O_2 in 50mM tris buffer. Sections were mounted on gelatin covered slides and dried overnight at 37C. Slides were dehydrated through a series of graded ethanol, xylene, and cover slipped with DPX.

Rodent Studies

All rodent animal procedures were performed in accordance with the University of Iowa animal care committee's regulations. Animals were housed in temperature-controlled conditions, provided food and water *ad libitum*, and maintained on a 12 h light/dark cycle (7:00 A.M. to 7:00 P.M.). Male C57BL/6J mice were purchased from The Jackson Laboratory. We first compared P7C3-A20 to NSI-189 for ability to increase the net magnitude of hippocampal neurogenesis in a standard 5 day *in vivo* assay of BrdU-labeled cells in the dentate gyrus (4). Here, mice are socially isolated without any environmental enrichment for 2 weeks, and then subjected to daily administration of both the test proneurogenic compound and a daily dose of BrdU (50 mg/kg ip). We next compared P7C3-A20 to NSI-189 in standard assays of proliferation and survival of newborn hippocampal neurons in the mouse dentate gyrus. Proliferation was assayed by measuring the number of BrdU+ cells / mm^3 dentate gyrus 1 hour after a pulse of BrdU (150 mg/kg ip) in animals that had received 3 days of daily P7C3-A20 (10 mg/kg/d ip) or NSI-189 (10 mg/kg/d ip). Next, to assay neuroprotective efficacy in promoting survival of newborn hippocampal neurons, we measured the number of BrdU+ cells / mm^3 dentate gyrus 15 days after a pulse of BrdU (150 mg/kg ip), in animals that had received daily treatment with either P7C3-A20 (10 mg/kg/d ip) or NSI-189 (10 mg/kg/d ip) starting at the same time as the delivery of the BrdU pulse. After BrdU (Sigma-Aldrich) administration, mice were euthanized at the specified time points by transcardial perfusion with 4% paraformaldehyde at pH 7.4, and brains were then processed for immunohistochemical detection of incorporated BrdU in the hippocampus. Dissected brains were immersed in 4% paraformaldehyde overnight at 4°C, and then cryoprotected in sucrose before being sectioned into 40-µm-thick free-floating sections. Unmasking of BrdU antigen was achieved through incubating tissue sections for 2 h in 50% formamide/2× saline-sodium citrate (SSC) at 65°C, followed by a 5 min wash in 2× SSC and subsequent incubation for 30 min in 2 M HCl at 37°C. Sections were processed for immunohistochemical staining with mouse monoclonal anti-BrdU (1:100, Roche). The number of BrdU+ cells in the entire dentate gyrus subgranular zone (SGZ) was quantified by counting BrdU+ cells within the SGZ and dentate gyrus in

every fifth section throughout the entire hippocampus, and then normalizing for dentate gyrus volume using Nikon Metamorph and NIH ImageJ software with appropriate conversion factors.

Results

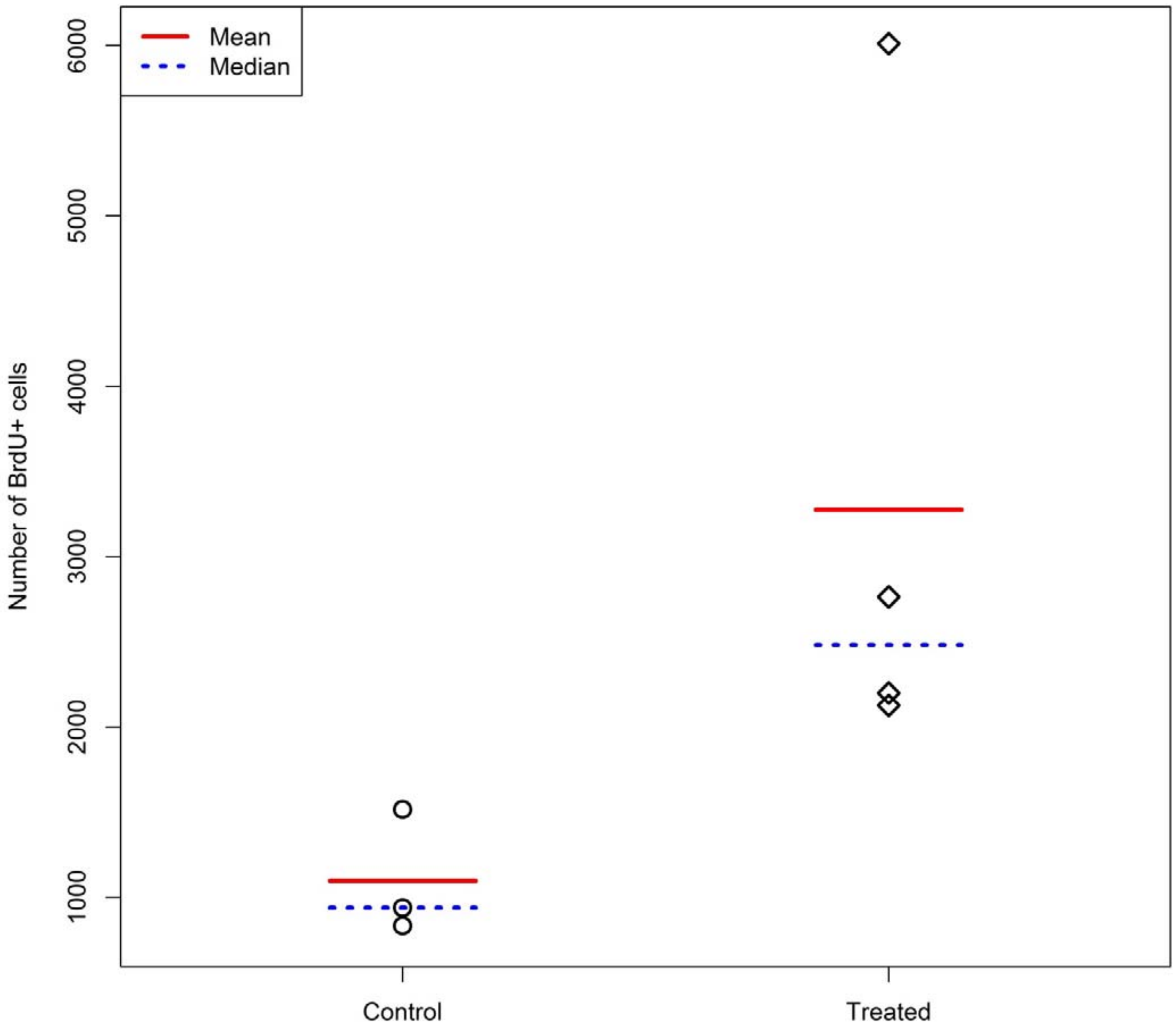

Supplemental Figure 1. Orally-Administered P7C3-A20 Elevates Survival of Newborn

The mean number of BrdU+ cells differed significantly ($t = -2.30$, $df = 3.31$, $p = 0.029$ based on permutation null distribution) between monkeys treated with P7C3-A20 (mean = 3275, SD = 1847) and those that received the vehicle control (mean = 1095, SD = 368). Although the median values in this relatively small sample size fell just short of significance based on a 0.05 threshold ($W = 0$, $p = 0.057$), all treated animals had higher numbers of BrdU+ cells than animals in the control group.

References

1. Yin TC, Britt JK, De Jesus-Cortes H, Lu Y, Genova RM, Khan MZ, et al. (2014): P7C3 neuroprotective chemicals block axonal degeneration and preserve function after traumatic brain injury. *Cell reports*. 8:1731-1740.
2. Lavenex P, Lavenex PB, Bennett JL, Amaral DG (2009): Postmortem changes in the neuroanatomical characteristics of the primate brain: hippocampal formation. *The Journal of comparative neurology*. 512:27-51.
3. Bauman MD, Amaral DG (2005): The distribution of serotonergic fibers in the macaque monkey amygdala: an immunohistochemical study using antisera to 5-hydroxytryptamine. *Neuroscience*. 136:193-203.
4. Pieper AA, Xie S, Capota E, Estill SJ, Zhong J, Long JM, et al. (2010): Discovery of a proneurogenic, neuroprotective chemical. *Cell*. 142:39-51.
